# Dual RNA-Seq Uncovers Metabolic Amino Acids Dependency of the Intracellular Bacterium *Piscirickettsia salmonis* Infecting Atlantic Salmon

**DOI:** 10.3389/fmicb.2018.02877

**Published:** 2018-11-27

**Authors:** Diego Valenzuela-Miranda, Cristian Gallardo-Escárate

**Affiliations:** Laboratory of Biotechnology and Aquatic Genomics, Interdisciplinary Center for Aquaculture Research, University of Concepción, Concepción, Chile

**Keywords:** dual RNA-seq, *Piscirickettsia salmonis*, Atlantic salmon, nutritional immunity, metabolic dependency, amino acids

## Abstract

High-throughput sequencing technologies have offered the possibility to understand the complexity of the transcriptomic responses of an organism during a wide variety of biological scenarios, such as the case of pathogenic infections. Recently, the simultaneous sequencing of both pathogen and host transcriptomes (dual RNA-seq) during the infection has become a promising approach to uncover the complexity of the host–pathogen interactions. In this study, through a double rRNA depletion and RNA sequencing protocols, we simultaneously analyzed the transcriptome of the intracellular bacterium *Piscirickettsia salmonis* and its host the Atlantic salmon (*Salmo salar*) during the course of the infection. Beyond canonical host immune-related response and pathogen virulent factors, both bacteria and host displayed a large number of genes associated with metabolism and particularly related with the amino acid metabolism. Notably, genome-wide comparison among *P. salmonis* genomes and different fish pathogens genomes revealed a lack of the biosynthetic pathway for several amino acids such as valine, leucine, and isoleucine. To support this finding, *in vitro* experiments evidenced that when these amino acids are restricted the bacterial growth dynamics is significantly affected. However, this condition is phenotypically reversed when the amino acids are supplemented in the bacterial growth medium. Based on our results, a metabolic dependency of *P. salmonis* on *S. salar* amino acids is suggested, which could imply novel mechanisms of pathogenesis based on the capacity to uptake nutrients from the host. Overall, dual transcriptome sequencing leads to the understanding of host–pathogen interactions from a different perspective, beyond biological processes related to immunity.

## Introduction

High-throughput sequencing technologies applied to transcriptomic studies (RNA-Seq) have offered the possibility to understand complex molecular responses under different biological scenarios. Among them, a pathogenic infection entails a deep transcriptomic remodeling of the host to promote the pathogenic clearance; in turn, pathogens display the expression of different genes to grant their survival and replicate within the host. In this context, the simultaneous analysis of host and pathogen transcriptomes (dual RNA-Seq) during their interaction can reveal novel aspects of the infective process (Westermann et al., 2017). Initially, this approach was limited to viral, fungal and parasitic infections, where the pathogen resembles host transcripts (Tierney et al., 2012; Strong et al., 2013; Choi et al., 2014; Pittman et al., 2014) and scarcely reported in bacterial models (Westermann et al., 2012). However, the improvement of high-throughput sequencing and the development of novel RNA capture/depletion methods offer a promising opportunity to also expand this approach to bacterial infections (Westermann et al., 2016).

Although most dual RNA-seq approaches applied in bacterial infections have been exploratory, some of them have unraveled novel mechanisms of host–pathogen interaction. For instance, a dual RNA-seq was used to discover a possible strategy employed by *Chlamydia trachomatis* for the *in vitro* infection of human epithelial cells based on an early iron acquisition and a host immune depletion strategy (Humphrys et al., 2013). Furthermore, the simultaneous transcriptome analysis of the Gram-negative bacterium *Haemophilus influenza* during the infection of mucosal epithelial cells revealed the importance of the host oxidative response and the mechanisms employed by the bacteria to overcome this adverse environment (Baddal et al., 2016). Likewise, the co-transcriptomic analysis of the uropathogenic *Escherichia coli* (UPEC) and its host evidenced that while host transcriptomic response was similar to different bacterial strains, different expression patterns were identified in UPEC strains with contrasting pathogenic effects (Mavromatis et al., 2015). These results were used to reveal novel insights into the mechanisms employed by the bacteria for the intra-macrophage survival. Moreover, a dual RNA-Seq was used to characterize the regulatory role of small RNAs (sRNAs) in *Salmonella enterica* infection. Here, researchers identified bacterial sRNA involved in the regulation of both host and pathogenic genes, revealing the hidden roles of *S. enterica* transcripts during the pathogenesis (Westermann et al., 2016). Simultaneous profiling of host-pathogen transcriptomes has become a powerful approach tool to unravel key aspects during the infection process. In the present study, we apply a dual RNA-Seq approach to reveal novel aspects of the infective process of the intracellular bacterium *Piscirickettsia salmonis* during the infection on the Atlantic salmon (*Salmo salar*).

*Piscirickettsia salmonis* is a facultative intracellular gram-negative bacterium that causes the salmonid rickettsial septicemia (SRS), a severe systemic disease responsible for up to 85% of the infectious moralities in farmed salmons in Chile. Just in this country, the economic losses associated with this pathogen has been estimated in around US$100 million per year (Smith et al., 1997; Bravo and Midtlyng, 2007; Pulgar et al., 2015), becoming one of the main concerns for the industry (Mauel and Miller, 2002). Beyond this negative impact, perhaps one of the most remarkable features of *P. salmonis* is its capability to infect and replicate within host immune cells, such as in macrophages (McCarthy et al., 2008; Rajas et al., 2009; Ramirez et al., 2015). In this context, the mechanism whereby this pathogen can evade host immune response are still unclear. Due to the high prevalence, negative impact and scientific interest on this pathogen, different efforts have tried to understand salmonids defensive mechanism against *P. salmonis* and how the bacteria overcome this response. Host transcriptomic response has been mainly associated with a regulation of genes involved in the innate immunity, apoptosis, different signaling pathways, endocytosis, non-coding RNAs and iron metabolism among others (Rise et al., 2004; Tacchi et al., 2011; Pulgar et al., 2015; Valenzuela-Miranda and Gallardo-Escarate, 2016; Valenzuela-Miranda et al., 2017). On the other hand, *P. salmonis* transcriptomic response has been assessed after the *in vitro* infection in Sf21 cell lines (Machuca and Martinez, 2016). Although different genes associated with the type IV secretion and iron acquisition system were identified, it remains unexplored how *P. salmonis* transcriptome is modulated during the infection. Due to this, we explore a dual RNA-Seq approach to unravel novel mechanisms of interactions during the infection of *P. salmonis* on the Atlantic salmon. A special emphasis was placed in bacterial gene expression, since transcriptional response of the Atlantic salmon against *P. salmonis* have been widely described previously (Tacchi et al., 2011; Pulgar et al., 2015; Valenzuela-Miranda and Gallardo-Escarate, 2016; Tarifeno-Saldivia et al., 2017). Beyond canonical pathogenic and immune related-genes, our results evidenced a common transcriptomic response between host and pathogen associated with the amino acid metabolism. Further analyses revealed a lack of *P. salmonis* genes associated with different amino acids biosynthetic pathways and the importance of the availability of some amino acids for the bacterial growth medium. We hypothesize metabolic amino acids dependency of *P. salmonis* on *S. salar*, which could imply novel mechanisms of pathogenesis based on the capacity to uptake nutrients from the host and capacity of the host to regulate the availability of free amino acids.

## Materials and Methods

### Experimental Design

Atlantic salmons (154.7 ± 13.5g) were obtained from a commercial farm located at Puerto Montt, Chile and transferred to the Marine Biology Station of the University of Concepción (Dichato, Chile). Here, individuals were randomly screened to discard the presence of different pathogens commonly present salmonid aquaculture. After quarantine, individuals were randomly divided into two independent marine water-based recirculating lines, each containing five 370 L tanks. For each line, three tanks were used for sampling (six in total), one tank was used to record mortality (two in total) and the remaining tank was not considered in this experiment. A total of 50 individuals per tank were maintained during an acclimation period of 14 days before the challenge. After this period, each individual was anesthetized and intraperitoneally injected with 0.2 ml of *P. salmonis* (EM-90 strain) containing 1 × 10^6^ bacteria per dose, as previously standardized. Later, samples were collected from infected individuals at 3, 7, and 14 days post infection (dpi). Head kidney and spleen tissues were collected from two individuals of each sampling tank (12 individuals per point) and stored in RNA later solution (Ambion, United States) at -80°C. In the remaining tanks, mortalities were daily recorded (Supplementary Figure S1), clinically and molecularly confirmed as a result of SRS. All animal procedures were carried out under the guidelines approved by the Ethics Committee of University of Concepción.

### RNA Isolation and Sequencing Strategy

Infected tissues stored at -80°C in RNA later solution were thawed at room temperature and total RNA (host and pathogen) was isolated from 10 different individuals using the TRIzol reagent kit (Thermo Fisher Scientific) according to manufactures instructions. RNA integrity was verified using the R6K screen tape 2200 on the TapeStation (Agilent Technologies, United States) platform. Thus, isolated RNAs with RNA Integrity Numbers (RIN) above 8 were considered for further analysis. Based on RNA quality, 3 different pools of RNA were prepared from 3 distinct individuals for each tissue and time (biological replicates). RNA pools were precipitated in absolute ethanol and shipped in dry ice to Macrogen Inc. (Korea). Here, two Ribo-Zero rRNA Removal Kit (Illumina, San Diego, CA, United States) were used to remove both bacterial and host rRNAs. Remaining RNA containing both *P. salmonis* and *S. salar* transcripts were used to prepare high-throughput sequencing libraries using the TrueSeq RNA sample preparation kit (Illumina, San Diego, CA, United States). Each library was sequenced on a HiSeq platform at 100 bp paired-end reads (Macrogen, Korea). All sequencing data will be available under the SRA accession number SUB4576220. Sequencing statistics for each RNA-seq data are presented in Supplementary Table S1.

### Dual RNA-Seq and Differential Expression Analysis

Raw sequencing reads were filtered by quality and adapter/index were identified and removed from remaining reads using CLC Genomics Workbench (V10, CLC Bio, Denmark). In order to discriminate pathogen and host transcriptomes, cleaned reads were mapped against the last available version of the Atlantic salmon (*S. salar)*^1^ and against *P. salmonis* available genomes^2^. Mapping parameters included a mismatch cost of 2, insertion/deletion cost of 3 and a similarity/length fraction of 0.8. Effectively mapped reads against both genomes were separated in different files and used for further RNA-Seq analysis. RNA-Seq analysis was conducted using CLC Genomics Workbench (V10, CLC Bio, Denmark). Previously discriminated reads from host and pathogen were used to perform RNA-Seq analysis using all coding sequences annotated in the Atlantic salmon and *P. salmonis* genomes. For RNA-Seq analyses, similarity/length fraction was set as 0.9 in order to minimize the probability to include misassigned reads for each species. Expression values were estimated as transcripts per million (TPM) and normalized by totals per million read. Expression values obtained at 3 dpi were used as baseline for gene expression comparison. We decided to use this dataset as reference because we needed transcriptomic data that contained reads from the pathogen to compare, since pathogenic reads in any type of control would not be present. Further, statistical differences were identified through a Baggerly’s test adjusting *p*-values through a false discovery rate (FDR) correction. Genes with a fold change > 4 and FDR *p*-values < 0.01 were considered as differentially expressed.

### Functional Annotation and qPCR Validations

Molecular annotation of the differentially expressed transcripts for both *P. salmonis* and *S. salar* was carried out to identify the most representative biological processes. For this purpose, the Gene Ontology (GO) annotation was conducted through the BLAST2GO software V 4.1.9 (Conesa et al., 2005) and the enrichment analysis was performed using as reference the genomes of *P. salmonis* and *S. salar*. Further, resulting GO enrichment analysis was visualized in REVIGO platform (Supek et al., 2011). Finally, KEGG pathway annotation analysis was also conducted using the KEGG automatic annotation server (Moriya et al., 2007) through the bidirectional best-hit assignment method. Furthermore, RT-qPCR were used to validate sequencing results. To do this, 10 genes from the bacteria and 10 genes from the fish were randomly selected and used to RT-qPCR primer design (Supplementary Table S2). After primer validation, each RT-qPCR was conducted in a thermocycler StepOne plus (Applied Biosystems, United States) using the Maxima SYBR Green/ROX kit according to manufactures instructions. Amplification cycles were used as following 95°C for 10 min, 40 cycles at 95°C for 30 s, 60°C for 30 s, and 72°C for 30 s. All qPCRs were carried on five biological and three technical replicates and expression values were estimated through the 2Δ Ct method using 16s and elongation factor 1a as normalizer genes for *P. salmonis* and *S. salar*, respectively. Significant differences between 7 and 14 dpi regarding 3 dpi were estimated with the Student’s *t*-test (*p* < 0.05). A comparison between fold-changes obtained through RT-qPCR and RNA-seq evidence a *r*^2^ value above 0.8, evidencing a high correlation between the fold changes obtained by RT-qPCR and RNA-seq (Supplementary Figure S2). Individual fold changes were included as Supplementary Figure S3.

### Exploring the Amino Acid Metabolism of *P. salmonis*

Dual RNA-Seq analysis revealed a large number of genes differentially expressed associated with amino acid metabolism during the infection process. Due to this, we further explored the importance of amino acids in *P. salmonis* metabolism. First, a genome-scale comparison was conducted between *P. salmonis*, a second salmonid pathogen such as *Aeromonas salmonicida* and a closely related bacterium as *Francisella tularensis*. For this purpose, coding genes were obtained from NCBI for *P. salmonis* (see text footnote 2), *F. tularensis*^3^ and *A. salmonicida*^4^ genomes. All coding sequences were annotated through KAAS annotation server as described above, focusing our attention in Histidine metabolism (00340), valine, leucine and isoleucine degradation/biosynthesis (00280 and 00290), Arginine and proline metabolism (00330), Lysine biosynthesis/degradation (00300 and 00310), Cysteine and methionine metabolism (00270), Glycine, serine and threonine metabolism (00260), Phenylalanine, tyrosine and tryptophan biosynthesis (00400) and Alanine, aspartate and glutamate metabolism (00250). Furthermore, liquid cultures of *P. salmonis* were conducted considering different experimental culture media with distinct amino acid composition. A basal medium (BM) was prepared with Eugon (30.4 g/l) supplemented with FeCl_3_ (2 mM), a complete medium (CM) prepared with Eugon (30.4 g/l) supplemented with FeCl_3_ (2 mM) and Casamino acid (1%) and experimental cultures medias were prepared with BM + 1% of the desired amino acid (Valine, leucine, and Isoleucine). A bacterial inoculum previously obtained from CHSE-214 cells infected with *P. salmonis* at 90% of lysis was used as starting material for the growth of *P. salmonis* in CM medium. When the exponential phase was reached, 300 μL from the liquid culture was used to inoculate 2.7 ml of liquid culture containing the different experimental mediums. All cultures were carried on triplicates and maintained at 20°C with a constant agitation of 100 rpm. Bacterial growth was daily based monitored through the change in the optical density at an absorbance of 600 nm. A multiple *t*-test was carried out to identify statistically significant differences (*p* < 0.01) between treatments.

## Results

### Exploring Host and Pathogen Transcriptome During Pathogenesis

**FIGURE 1:**
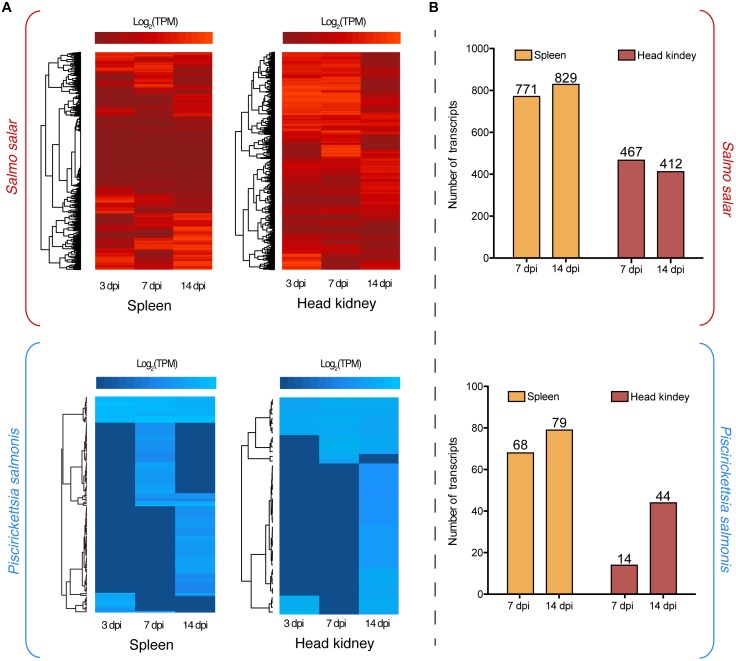
**(A)** Global transcriptome analysis of *Piscirickettsia salmoni*s (blue) and *S. salar* (red) at 3, 7, and 14 days post-infection (dpi) in spleen and head kidney tissues. Expression values were estimated as transcripts per million reads (TPM) and Log_2_ transformed for heat map clustering. **(B)** Differentially expressed genes in spleen and head kidney at 7 and 14 dpi using as control the expression values at 3 dpi. Three days post-infection were used as baseline gene expression since no bacterial reads would be present in any type of control group.

**FIGURE 2:**
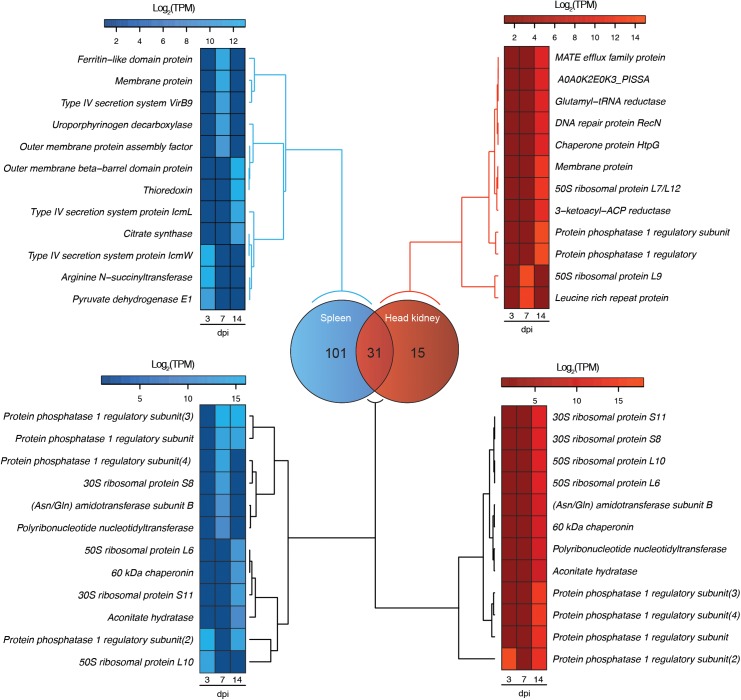
Venn diagram showing the number of exclusive and shared genes in the transcriptome of *P. salmonis* during the infection in spleen (blue) and head kidney (red) tissues of Atlantic salar. The heat maps show a subset of exclusive and shared genes expressed by *P. salmonis* in both tissues.

Dual RNA-Seq analysis evidenced the modulation of *P. salmonis* and *S. salar* transcriptomes during the infection. This modulation is represented in two heat maps, one for the fish host (red) and another one for the pathogen (blue), where different clusters of expression profiles were identified (Figure 1A). Regarding *S. salar* transcriptome, 771 and 829 genes were differentially expressed in spleen at 7 and 14 dpi, respectively (Figure 1B). Meanwhile, 412 and 467 genes were differentially modulated in the head kidney at the same time-points (Figure 1B). On the other hand, 68 and 79 *P. salmonis* genes were differentially expressed in the spleen at 7 and 14 days, respectively, while 14 and 44 were identified in the head kidney (Figure 1B). Thus, the number of bacterial genes differentially expressed were increased together with the course of the infection. Since transcriptional responses for the host have been previously reported, a special focus was placed on bacterial gene expression. Herein, a Venn diagram analysis revealed that 31 *P. salmonis* genes were differentially regulated in both spleen and head kidney, while 101 and 15 transcripts were exclusively regulated in spleen and head kidney, respectively. Genes exclusively regulated in spleen included a *ferritin-like domain protein*, genes associated with the type IV secretion system (*VirB9, IcmL*, and *IcmW*) and several outer membrane proteins (Figure 2). On the other hand, the genes exclusively regulated in head kidney included a *MATE efflux family protein, DNA repair protein RecN, chaperone protein HtpG* and membrane proteins among others (Figure 2). Among shared genes, different Protein phosphatase 1, ribosomal protein, chaperones and Asn/Gln aminotransferase subunits were also found (Figure 2). The complete list of differentially expressed genes for the Atlantic salmon and *P. salmonis* is included as Supplementary Table S3.

### Amino Acid Metabolism: A Common Response Between *P. salmonis* and *S. salar*

**FIGURE 3:**
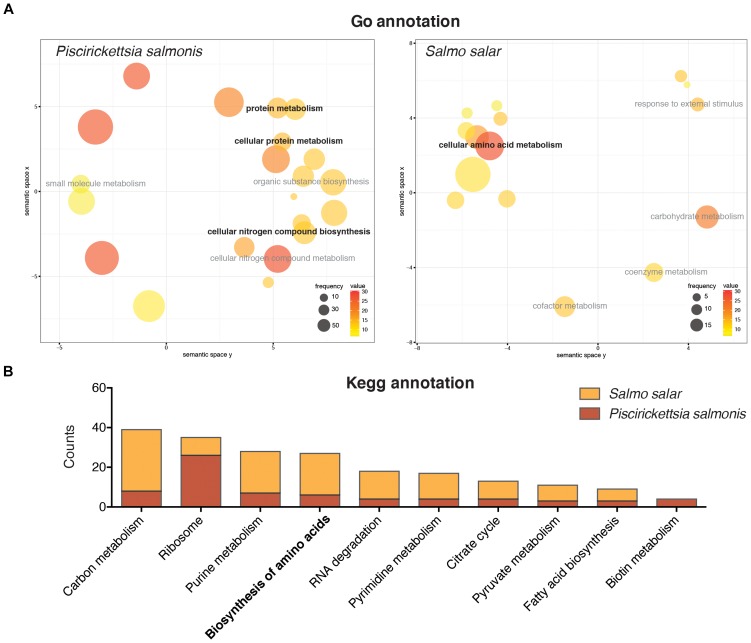
Gene ontology annotation **(A)** and KEGG pathway annotation **(B)** of the differentially expressed genes in *Salmo salar* and *P. salmonis* during the infection. The size of each circle represents the frequency of the GO term in the underlying GO database (larger circles represent more general terms) and the color represents the number of genes in each term (value). Both host and pathogen displayed a large number of genes associated to amino acid biosynthesis and degradation.

A functional annotation of the different differentially expressed genes for both *S. salar* and *P. salmonis* was conducted in order to identify key molecular pathways regulated during the infective process. GO enrichment analysis evidenced that a large percent of differentially expressed genes in *P. salmonis* belonged to biological processes related with the metabolism of proteins and nitrogen compounds, such as the terms “protein metabolism,” “cellular protein metabolism” and “cellular nitrogen compound biosynthesis” among others (Figure 3A). On the other hand, a more complex transcriptomic response was found in Atlantic salmon. Here, multiple biological processes were represented, such as organic acid metabolic process, oxidation-reduction process, response to external stimulus, chemotaxis among others (Supplementary Table S2). However, terms related to the metabolism of amino acids was also represented within differentially expressed transcripts, such as the “cellular amino acid metabolism” (Figure 3A). Enrichment of genes associated with protein metabolism was also found through KEGG annotation. Furthermore, one of the most represented pathways among differentially regulated genes in *P. salmonis* transcriptome included different metabolic pathways, among them, the “biosynthesis and degradation of amino acids” (Figure 3B). Regarding host transcriptome, the response of the Atlantic salmon was associated not just associated with endocytosis, cytokine-cytokine receptor interaction, apoptosis, phagosome and Nod-like receptor signaling pathways (Supplementary Table S4), but also with key metabolic pathways, including the “biosynthesis of amino acids” (Figure 3B). Overall, the results evidenced that although the metabolism of amino acids was not the predominant transcriptomic response in the host, both *P. salmonis* and *S. salar* displayed a large number of genes involved with biosynthesis and degradation of amino acids. Due to this common response, we further investigate the role of amino acids in *P. salmonis* metabolism.

### Role of Amino Acids in *P. salmonis* Metabolism

**FIGURE 4:**
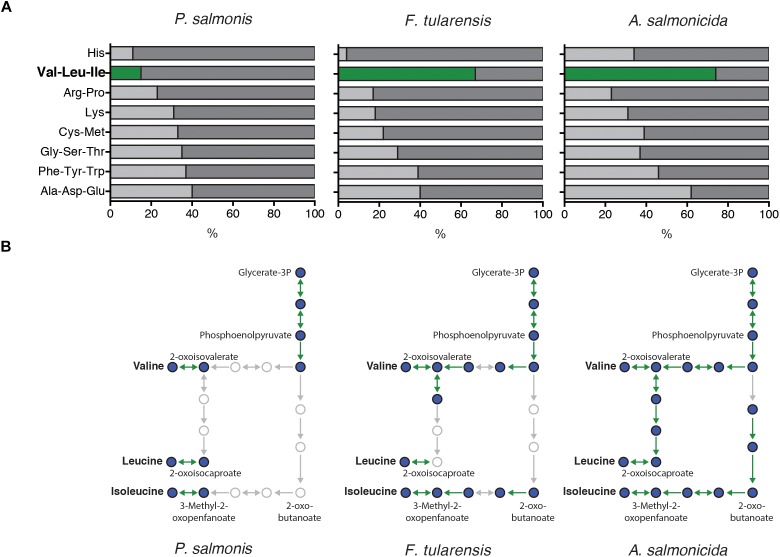
**(A)** Genomic-wide comparison of genes involved in the biosynthesis and degradation of amino acid in *P. salmonis*, a close related bacterium (*F. tularensis*) and another fish Gram negative bacterium (*A. salmonicida*). Bars represent the percentage of genes identified for each pathogen underlying all the possible genes for the pathway in KEGG pathway database. **(B)** Subset of the biosynthesis and degradation pathway for valine, leucine, and isoleucine in *P. salmonis, F. tularensis*, and *A. salmonicida*. Circles represent intermediate molecules and arrows the genes catalyzing each reaction. Thus, a green arrow represents a gene that was found in the genome of each pathogen and gray ones for those absent. Thus, *P. salmonis* evidenced a smaller number of genes involved in valine, leucine, and isoleucine biosynthesis/degradation.

**FIGURE 5:**
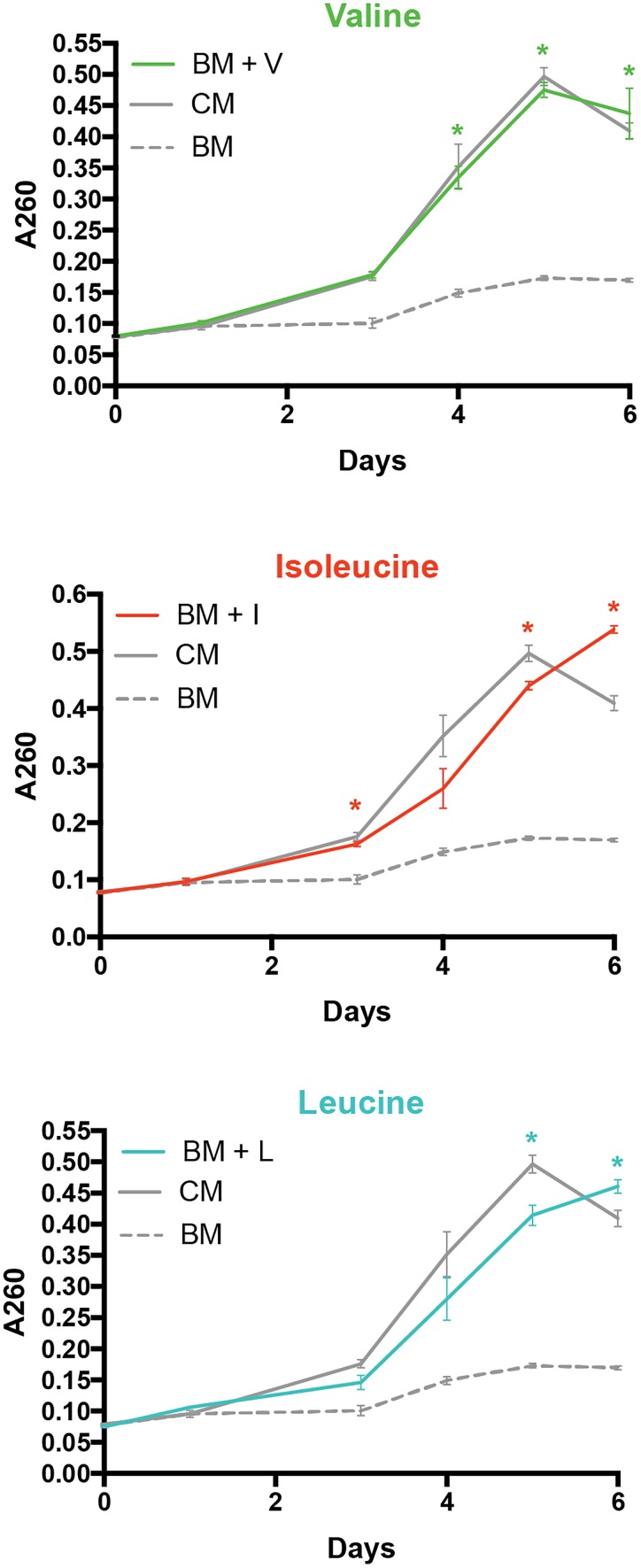
Growth kinetics of *P. salmonis* in liquid culture media supplemented with different amino acids. Bacteria was growth in a control medium (CM), a basal medium without amino acids (BM) and in basal medium supplemented with valine (BM + V), leucine (BM + L), and isoleucine (BM + I) amino acids at 1%. ^∗^Represents statistically significant differences (*p* < 0.01) between the supplemented medium (Val, Leu, or Ile) regarding control group (no amino acids).

To explore the importance of amino acids in *P. salmonis* metabolism, a genome-scale comparison was used to identify the presence/absence of genes directly involved in the degradation/biosynthesis of different bacterial pathogens. Thus, *P. salmonis* genome was compared with *F. tularensis* and *A. salmonicida* available genomes. Results showed that all three pathogens dispose of a similar genetic background of genes involved in biosynthesis/degradation of amino acids (Figure 4). However, a significant lack of genes related with valine, leucine, and isoleucine metabolism was found in *P. salmonis* compared with *F. tularensis* and *A. salmonicida*, where over 60% of all possible genes were found (Figure 4A). However, a deeper look into this metabolic pathway evidenced that although *P. salmonis* lacks the majority of the genetic background for the biosynthesis of this amino acids, it was possible to found the gene that encodes for the primary degradation of valine, leucine, and isoleucine (Figure 4B). These results suggest that the bacterium is not able to biosynthesize these amino acids and therefore a metabolic dependency of *P. salmonis* on environmental host amino acid availability can be expected. To further explore the importance of amino acid availability to the bacteria, *P. salmonis* was growth in different liquid culture mediums with different amino acidic availability. A control medium (CM) fully supplemented with amino acids, a basal medium (BM) with no supplementation of amino acids and basal mediums supplemented with 1% of either valine (BM + V), leucine (BM + I) or isoleucine (BM + L) were used as experimental mediums. The results evidence that the bacteria are not able to grow when no amino acid is supplemented. However, when BM is supplemented with either valine, leucine or isoleucine, the growth kinetics of *P. salmonis* resembles the one observed in a fully amino acidic supplemented condition (Figure 5).

## Discussion

Dual RNA-Seq has emerged as a promising approach to elucidate host–pathogen interaction. Although this approach was previously limited to pathogens that resembles host transcripts (Westermann et al., 2017), the development of high-throughput sequencing technologies has allowed to expand this approach to bacterial infections. In this context, we applied a dual RNA-Seq approach to explore novel means of interaction between the intracellular bacterium *P. salmonis* and its main host the Atlantic salmon (*S. salar*). In addition to being the main threat to Chilean salmonid aquaculture (Rozas and Enriquez, 2014), the intracellular nature of this pathogen makes it an interesting model to study host immune evasion strategies and intracellular survival mechanisms employed by intracellular pathogens.

The dual RNA-Seq analysis revealed the presence of different bacterial genes among the spleen and head kidney transcriptome. Thus, the spleen showed the largest number of bacterial genes regarding the ones found in head kidney data. This can be attributable due to when IP injection is used as infection method in previous IP challenges with *P. salmonis*, the spleen is one of the first tissues to be infected, followed by head kidney (Valenzuela-Miranda and Gallardo-Escarate, 2016). Therefore, the differences between the transcriptional modulation of pathogen genes among this tissue could be as a result of different infection stages rather than a tissue-specific transcriptomic response of the pathogen. Nevertheless, different classical pathogenic related genes were identified in transcriptomic data. Such as the case of several members of the type IV secretion system, including *VirB9, IcmL*, and *IcmW*. The type IV secretion system it has been described as a conserved mechanism for the delivery of virulent factors from host to pathogen (Thanassi and Hultgren, 2000). This system has been previously described in *P. salmonis* (Gomez et al., 2013) and even the directed mutagenesis of this locus has resulted in the attenuation of the pathogenesis of *P. salmonis* (Mancilla et al., 2018). On the other hand, the repertory of genes displayed by the Atlantic salmon in response to infection was classified into different molecular pathways. These processes included endocytosis, cytokine-cytokine receptor interaction, apoptosis, phagosome and Nod-like receptor signaling pathways, which has been previously described as key responses triggered during the infection of *P. salmonis* in *S. salar* tissues (Valenzuela-Miranda and Gallardo-Escarate, 2016).

However, beyond the canonical pathogenic genes commonly associated with bacterial pathogenesis and the Atlantic salmon immunity (Valenzuela-Miranda and Gallardo-Escarate, 2016; Tarifeno-Saldivia et al., 2017), a common response associated with protein metabolism and particularly the biosynthesis/degradation of amino acids was present in both *S. salar* and *P. salmonis* transcriptomic response during the infection process. Due to this, a genome-scale comparison was performed in order to evidence the genetic background of genes involved in biosynthesis degradation of amino acids in *P. salmonis*, a close related bacterium *F. tularensis* and another salmonid bacterial pathogen *A. salmonicida*. The genomic background has been previously used to predict essential and non-essential amino acids in different pathogens (Meibom and Charbit, 2010). Based on our results, we found a lack of biosynthetic genes associated with the metabolism of valine, leucine, and isoleucine for *P. salmonis* when compared with *F. tularensis* and *A. salmonicida* genomes. Therefore, the availability of these amino acids for *P. salmonis* might rely upon the presence of these resources in the host intracellular environment.

During infection, intracellular pathogens must overcome different adverse condition, such as the entrance to host cells, host immune response, free radicals and also nutrient deprivation. Although host cytosol was previously considered as an abundant source of nutrients for invading pathogens (Ray et al., 2009), recent evidence suggests that hosts can reduce the intracellular availability of certain nutrients as a protective response against the invading pathogens (Abu Kwaik and Bumann, 2013; Barel and Charbit, 2013). This deprivation results in a struggle between host and pathogen for the limited nutrient availability, which commonly known as nutritional immunity (Hood and Skaar, 2012). Thus, it has been suggested that part of the immune response of the Atlantic salmon to *P. salmonis* infection relies on the nutritional immunity. This has been exclusively explored due to the struggle for iron availability. From *P. salmonis* perspective, it has been suggested the importance of a siderophore-based mechanism to capture iron from different host sources (Calquin et al., 2018). On the other hand, the infection of *P. salmonis* induces a strong transcriptomic modulation of genes involved in iron availability in *S. salar* (Pulgar et al., 2015; Valenzuela-Miranda and Gallardo-Escarate, 2016). In this context, our results evidence that both host and pathogen display a large number of genes involved in the biosynthesis/degradation of amino acids. Considering the lack of biosynthetic pathways in leucine, valine and isoleucine in *P. salmonis* and that these same amino acids are defined as essential amino acids for salmonids (Helland et al., 2010), we suggest that this transcriptional modulation can be reflecting an amino acid-based nutritional immunity triggered by *S. salar* to overcome *P. salmonis* infection. In turn, *P. salmonis* displayed a transcriptional modulation of different genes associated with amino acid metabolism to deal with host response.

Intracellular pathogens have developed different strategies to overcome amino acids starvation triggered by the host during infection. Some of these adaptations include the growth arrest and differentiation, an amino acid self-sufficiency and to exploit host machinery to obtain amino acids form host cell (Zhang and Rubin, 2013). Based on our results and considering the recent reconstruction of metabolic models for the bacteria (Cortes et al., 2017), the self-sufficiency strategy for amino acids in the *P. salmonis* can be rejected. Regarding growth arrest and differentiation, it has been reported that during an amino acid restricted scenario *C. trachomatis* morphologically changes into an aberrant form that is unable to grow but protects them from a nutrient restricted environment (Leonhardt et al., 2007). However, the addition of tryptophan or isoleucine can restore these aberrant forms and reactivate bacterial growth (Hatch, 1975; Ibana et al., 2011). Previously, it has been reported the existence of morphological small variants of *P. salmonis*, which were suggested as a survival mechanism employed by the bacteria to overcome adverse scenarios (Veronica Rojas et al., 2008). However, the role of these variants during an amino acid restricted scenario and its relation with virulence remains unexplored. On the other hand, pathogens like *Legionella pneumophila* have devolved mechanism that grants the access to host nutrients by promoting the expression of host amino acid transporters and taking advantage of proteasomes of infected cells to generate free amino acids for bacterial growth (Wieland et al., 2005; Price et al., 2011). In this context, it has been suggested that one of the mechanisms employed by *P. salmonis* to evade immune response can be related with the regulation of host transcriptional response through non-coding RNAs (Valenzuela-Miranda and Gallardo-Escarate, 2016; Valenzuela-Miranda et al., 2017), therefore, the idea that *P. salmonis* is able to hijack host machinery to obtain amino acids should also be explored.

The importance of amino acids in pathogenic cell cycle has not only be associated as a primary resource for the biogenesis of proteins, but also as alternative sources of carbon and nitrogen (Steeb et al., 2013). Therefore, a metabolic plasticity to obtain carbon and energy from multiple amino acid sources could become an advantage in a resource-limited scenario. This metabolic plasticity has been reported in other intracellular pathogens like *F. tularensis*, where the intracellular survival of this bacteria relies on their availability to exploit multiple host amino acids (Meibom and Charbit, 2010; Barel et al., 2015). Based in our results, and considering the recently proposed metabolic models for *P. salmonis* (Cortes et al., 2017; Fuentealba et al., 2017), we can hypothesize that this bacteria is capable to use different amino acids as a carbon an energy resource. This strategy has been adopted by others intracellular bacterium, where this capability has been described as a crucial factor for virulence development (Eisenreich et al., 2010; Meibom and Charbit, 2010; Barel et al., 2015). In this scenario, the implications of a metabolic plasticity in *P. salmonis* and its link to virulence of different bacterial strains must be further explored.

Overall, our results showed how a dual RNA-Seq approach can lead us to the understanding of novel means of interaction between host and pathogens. However, the importance of an amino acid-based nutritional immunity of *S. salar* in response to *P. salmonis* infection must be further investigated. This information will not just lead us to the development of novel treatments for the pathogen, but also to the understanding of the pathogenesis process from a different perspective, beyond canonical immunological mechanisms.

## Author Contributions

DV-M and CG-E conceived the study and drafted the manuscript. DV-M performed the experiments and analyzed the data under CG-E extensive supervision.

## Conflict of Interest Statement

The authors declare that the research was conducted in the absence of any commercial or financial relationships that could be construed as a potential conflict of interest.

## Footnotes

1 https://www.ncbi.nlm.nih.gov/assembly/GCF_000233375.1/

2 https://www.ncbi.nlm.nih.gov/genome/genomes/11769

3 https://www.ncbi.nlm.nih.gov/genome/genomes/511

4 https://www.ncbi.nlm.nih.gov/genome/genomes/540

## References

[B1] Abu Kwaik Y. Bumann D. (2013). Microbial quest for food in vivo: ‘nutritional virulence’ as an emerging paradigm. *Cell. Microbiol.* 15 882–890. 10.1111/cmi.12138 23490329

[B2] Baddal B. Muzzi A. Censini S. Calogero R. A. Torricelli G. Guidotti S. (2016). Erratum for baddal et al., dual RNA-seq of nontypeable *Haemophilus influenzae* and host cell transcriptomes reveals novel insights into host-pathogen cross talk. *Mbio* 7:e003 73–16. 26578681 10.1128/mBio.01765-15 PMC4659474

[B3] Barel M. Charbit A. (2013). *Francisella tularensis* intracellular survival: to eat or to die. *Microb. Infect.* 15 989–997. 10.1016/j.micinf.2013.09.009 24513705

[B4] Barel M. Ramond E. Gesbert G. Charbit A. (2015). The complex amino acid diet of francisella in infected macrophages. *Front. Cell. Infect. Microbiol.* 5:9. 10.3389/fcimb.2015.00009 25705612 PMC4319460

[B5] Bravo S. Midtlyng P. J. (2007). The use of fish vaccines in the chilean salmon industry 1999-2003. *Aquaculture* 270 36–42.

[B6] Calquin P. Ruiz P. Oliver C. Sanchez P. Haro R. Oliva H. (2018). Physiological evidence that *Piscirickettsia salmonis* produces siderophores and uses iron from different sources. *J. Fish Dis.* 41 553–558. 29193147 10.1111/jfd.12745

[B7] Choi Y. J. Aliota M. T. Mayhew G. F. Erickson S. M. Christensen B. M. (2014). Dual RNA-seq of parasite and host reveals gene expression dynamics during filarial worm-mosquito interactions. *PLoS Negl. Trop. Dis.* 8:e2905. 10.1371/journal.pntd.0002905 24853112 PMC4031193

[B8] Conesa A. Gotz S. Garcia-Gomez J. M. Terol J. Talon M. Robles M. (2005). Blast2GO: a universal tool for annotation, visualization and analysis in functional genomics research. *Bioinformatics.* 21 3674–3676. 16081474 10.1093/bioinformatics/bti610

[B9] Cortes M. P. Mendoza S. N. Travisany D. Gaete A. Siegel A. Cambiazo V. (2017). Analysis of *Piscirickettsia salmonis* metabolism using genome-scale reconstruction, modeling, and testing. *Front. Microbiol.* 8:2462. 10.3389/fmicb.2017.02462 29321769 PMC5732189

[B10] Eisenreich W. Dandekar T. Heesemann J. Goebel W. (2010). Carbon metabolism of intracellular bacterial pathogens and possible links to virulence. *Nat. Rev. Microbiol.* 8 401–412. 10.1038/nrmicro2351 20453875

[B11] Fuentealba P. Aros C. Latorre Y. Martinez I. Marshall S. Ferrer P. (2017). Genome-scale metabolic reconstruction for the insidious bacterium in aquaculture *Piscirickettsia salmonis*. *Bioresour. Technol.* 223 105–114. 10.1016/j.biortech.2016.10.024 27788423

[B12] Gomez F. A. Tobar J. A. Henriquez V. Sola M. Altamirano C. Marshall S. H. (2013). Evidence of the presence of a functional Dot/Icm type IV-B secretion system in the fish bacterial pathogen *Piscirickettsia salmonis*. *PLoS One* 8:e54934. 10.1371/journal.pone.0054934 23383004 PMC3557282

[B13] Hatch T. P. (1975). Competition between *Chlamydia psittaci* and L cells for host isoleucine pools: a limiting factor in chlamydial multiplication. *Infect. Immun.* 12 211–220. 1095493 10.1128/iai.12.1.211-220.1975 PMC415269

[B14] Helland S. J. Hatlen B. Grisdale-Helland B. (2010). Energy, protein and amino acid requirements for maintenance and efficiency of utilization for growth of atlantic salmon post-smolts determined using increasing ration levels. *Aquaculture* 305 150–158.

[B15] Hood M. I. Skaar E. P. (2012). Nutritional immunity: transition metals at the pathogen-host interface. *Nat. Rev. Microbiol.* 10 525–537. 10.1038/nrmicro2836 22796883 PMC3875331

[B16] Humphrys M. S. Creasy T. Sun Y. Shetty A. C. Chibucos M. C. Drabek E. F. (2013). Simultaneous transcriptional profiling of bacteria and their host cells. *PLoS One* 8:e80597. 10.1371/journal.pone.0080597 24324615 PMC3851178

[B17] Ibana J. A. Belland R. J. Zea A. H. Schust D. J. Nagamatsu T. AbdelRahman Y. M. (2011). Inhibition of indoleamine 2,3-dioxygenase activity by levo-1-methyl tryptophan blocks gamma interferon-induced *Chlamydia trachomatis* persistence in human epithelial cells. *Infect. Immun.* 79 4425–4437. 10.1128/IAI.05659-11 21911470 PMC3257928

[B18] Leonhardt R. M. Lee S. J. Kavathas P. B. Cresswell P. (2007). Severe tryptophan starvation blocks onset of conventional persistence and reduces reactivation of *Chlamydia trachomatis*. *Infect. Immun.* 75 5105–5117. 17724071 10.1128/IAI.00668-07 PMC2168275

[B19] Machuca A. Martinez V. (2016). Transcriptome analysis of the intracellular facultative pathogen *Piscirickettsia salmonis*: expression of putative groups of genes associated with virulence and iron metabolism. *PLoS One* 11:e0168855. 10.1371/journal.pone.0168855 28033422 PMC5199080

[B20] Mancilla M. Saavedra J. Grandon M. Tapia E. Navas E. Grothusen H. (2018). The mutagenesis of a type IV secretion system locus of *Piscirickettsia salmonis* leads to the attenuation of the pathogen in atlantic salmon, Salmo salar. *J. Fish Dis.* 41 625–634. 10.1111/jfd.12762 29251345

[B21] Mauel M. J. Miller D. L. (2002). Piscirickettsiosis and piscirickettsiosis-like infections in fish: a review. *Vet. Microbiol.* 87 279–289. 12069766 10.1016/s0378-1135(02)00085-8

[B22] Mavromatis C. H. Bokil N. J. Totsika M. Kakkanat A. Schaale K. Cannistraci C. V. (2015). The co-transcriptome of uropathogenic *Escherichia coli*-infected mouse macrophages reveals new insights into host-pathogen interactions. *Cell. Microbiol.* 17 730–746. 10.1111/cmi.12397 25410299 PMC4950338

[B23] McCarthy U. M. Bron J. E. Brown L. Pourahmad F. Bricknell I. R. Thompson K. D. (2008). Survival and replication of *Piscirickettsia salmonis* in rainbow trout head kidney macrophages. *Fish Shellfish Immun.* 25 477–484. 10.1016/j.fsi.2008.07.005 18691656

[B24] Meibom K. L. Charbit A. (2010). *Francisella tularensis* metabolism and its relation to virulence. *Front. Microbiol.* 1:140 10.3389/fmicb.2010.00140 PMC3109416 21687763

[B25] Moriya Y. Itoh M. Okuda S. Yoshizawa A. C. Kanehisa M. (2007). KAAS: an automatic genome annotation and pathway reconstruction server. *Nucleic Acids Res.* 35 W182–W185. 17526522 10.1093/nar/gkm321 PMC1933193

[B26] Pittman K. J. Aliota M. T. Knoll L. J. (2014). Dual transcriptional profiling of mice and Toxoplasma gondii during acute and chronic infection. *BMC Genomics* 15:806. 10.1186/1471-2164-15-806 25240600 PMC4177681

[B27] Price C. T. Al-Quadan T. Santic M. Rosenshine I. Abu Kwaik Y. (2011). Host proteasomal degradation generates amino acids essential for intracellular bacterial growth. *Science* 334 1553–1557. 10.1126/science.1212868 22096100

[B28] Pulgar R. Hodar C. Travisany D. Zuniga A. Dominguez C. Maass A. (2015). Transcriptional response of atlantic salmon families to *Piscirickettsia salmonis* infection highlights the relevance of the iron-deprivation defence system. *BMC Genomics* 16:495. 10.1186/s12864-015-1716-9 26141111 PMC4490697

[B29] Rajas V. Galanti N. Bols N. C. Marshall S. H. (2009). Productive Infection of *Piscirickettsia salmonis* in macrophages and monocyte-like cells from rainbow trout, a possible survival strategy. *J. Cell. Biochem.* 108 631–637. 10.1002/jcb.22295 19681041

[B30] Ramirez R. Gomez F. A. Marshall S. H. (2015). The infection process of *Piscirickettsia salmonis* in fish macrophages is dependent upon interaction with host-cell clathrin and actin. *FEMS Microbiol. Lett.* 362 1–8. 10.1093/femsle/fnu012 25790493

[B31] Ray K. Marteyn B. Sansonetti P. J. Tang C. M. (2009). Life on the inside: the intracellular lifestyle of cytosolic bacteria. *Nat. Rev. Microbiol.* 7 333–340. 10.1038/nrmicro2112 19369949

[B32] Rise M. L. Jones S. R. Brown G. D. von Schalburg K. R. Davidson W. S. Koop B. F. (2004). Microarray analyses identify molecular biomarkers of atlantic salmon macrophage and hematopoietic kidney response to *Piscirickettsia salmonis* infection. *Physiol. Genomics* 20 21–35. 15454580 10.1152/physiolgenomics.00036.2004

[B33] Rozas M. Enriquez R. (2014). Piscirickettsiosis and *Piscirickettsia salmonis* in fish: a review. *J. Fish Dis.* 37 163–188. 10.1111/jfd.12211 24279295

[B34] Smith P. A. Contreras J. R. Larenas J. J. Aguillon J. C. Garces L. H. Perez B. (1997). Immunization with bacterial antigens: piscirickettsiosis. *Dev. Biol.* 90 161–166. 9270845

[B35] Steeb B. Claudi B. Burton N. A. Tienz P. Schmidt A. Farhan H. (2013). Parallel exploitation of diverse host nutrients enhances *Salmonella* virulence. *PLoS Pathog.* 9:e1003301. 10.1371/journal.ppat.1003301 23633950 PMC3636032

[B36] Strong M. J. Xu G. Coco J. Baribault C. Vinay D. S. Lacey M. R. (2013). Differences in gastric carcinoma microenvironment stratify according to EBV infection intensity: implications for possible immune adjuvant therapy. *PLoS Pathog.* 9:e1003341. 10.1371/journal.ppat.1003341 23671415 PMC3649992

[B37] Supek F. Bosnjak M. Skunca N. Smuc T. (2011). REVIGO summarizes and visualizes long lists of gene ontology terms. *PLoS One* 6:e21800. 10.1371/journal.pone.0021800 21789182 PMC3138752

[B38] Tacchi L. Bron J. E. Taggart J. B. Secombes C. J. Bickerdike R. Adler M. A. (2011). Multiple tissue transcriptomic responses to *Piscirickettsia salmonis* in atlantic salmon (Salmo salar). *Physiol. Genomics* 43 1241–1254. 10.1152/physiolgenomics.00086.2011 21878610

[B39] Tarifeno-Saldivia E. Valenzuela-Miranda D. Gallardo-Escarate C. (2017). In the shadow: the emerging role of long non-coding RNAs in the immune response of atlantic salmon. *Dev. Comp. Immunol.* 73 193–205. 10.1016/j.dci.2017.03.024 28373064

[B40] Thanassi D. G. Hultgren S. J. (2000). Multiple pathways allow protein secretion across the bacterial outer membrane. *Curr. Opin. Cell Biol.* 12 420–430. 10873830 10.1016/s0955-0674(00)00111-3

[B41] Tierney L. Linde J. Muller S. Brunke S. Molina J. C. Hube B. (2012). An interspecies regulatory network inferred from simultaneous RNA-seq of candida albicans invading innate immune cells. *Front. Microbiol.* 3:85. 10.3389/fmicb.2012.00085 22416242 PMC3299011

[B42] Valenzuela-Miranda D. Gallardo-Escarate C. (2016). Novel insights into the response of atlantic salmon (Salmo salar) to *Piscirickettsia salmonis*: interplay of coding genes and lncRNAs during bacterial infection. *Fish Shellfish Immunol.* 59 427–438. 10.1016/j.fsi.2016.11.001 27818337

[B43] Valenzuela-Miranda D. Valenzuela-Munoz V. Farlora R. Gallardo-Escarate C. (2017). MicroRNA-based transcriptomic responses of atlantic salmon during infection by the intracellular bacterium *Piscirickettsia salmonis*. *Dev. Comp. Immunol.* 77 287–296. 10.1016/j.dci.2017.08.016 28870451

[B44] Veronica Rojas M. Olivares P. J. del Rio R. Marshall S. H. (2008). Characterization of a novel and genetically different small infective variant of *Piscirickettsia salmonis*. *Microb. Pathog.* 44 370–378. 10.1016/j.micpath.2007.10.012 18166333

[B45] Westermann A. J. Barquist L. Vogel J. (2017). Resolving host-pathogen interactions by dual RNA-seq. *PLoS Pathog.* 13:e1006033. 10.1371/journal.ppat.1006033 28207848 PMC5313147

[B46] Westermann A. J. Forstner K. U. Amman F. Barquist L. Chao Y. Schulte L. N. (2016). Dual RNA-seq unveils noncoding RNA functions in host-pathogen interactions. *Nature* 529 496–501. 10.1038/nature16547 26789254

[B47] Westermann A. J. Gorski S. A. Vogel J. (2012). Dual RNA-seq of pathogen and host. *Nat. Rev. Microbiol.* 10 618–630. 22890146 10.1038/nrmicro2852

[B48] Wieland H. Ullrich S. Lang F. Neumeister B. (2005). Intracellular multiplication of *Legionella pneumophila* depends on host cell amino acid transporter SLC1A5. *Mol. Microbiol.* 55 1528–1537. 15720558 10.1111/j.1365-2958.2005.04490.x

[B49] Zhang Y. J. Rubin E. J. (2013). Feast or famine: the host-pathogen battle over amino acids. *Cell Microbiol.* 15 1079–1087. 10.1111/cmi.12140 23521858 PMC6434321

