# Supplementary figures and images for: Dual RNA-Seq Uncovers Metabolic Amino Acids Dependency of the Intracellular Bacterium Piscirickettsia salmonis Infecting Atlantic Salmon
Source: Front Microbiol. 2018 Nov 27;9:2877. doi: 10.3389/fmicb.2018.02877 (PMC6277808; doi:10.3389/fmicb.2018.02877)

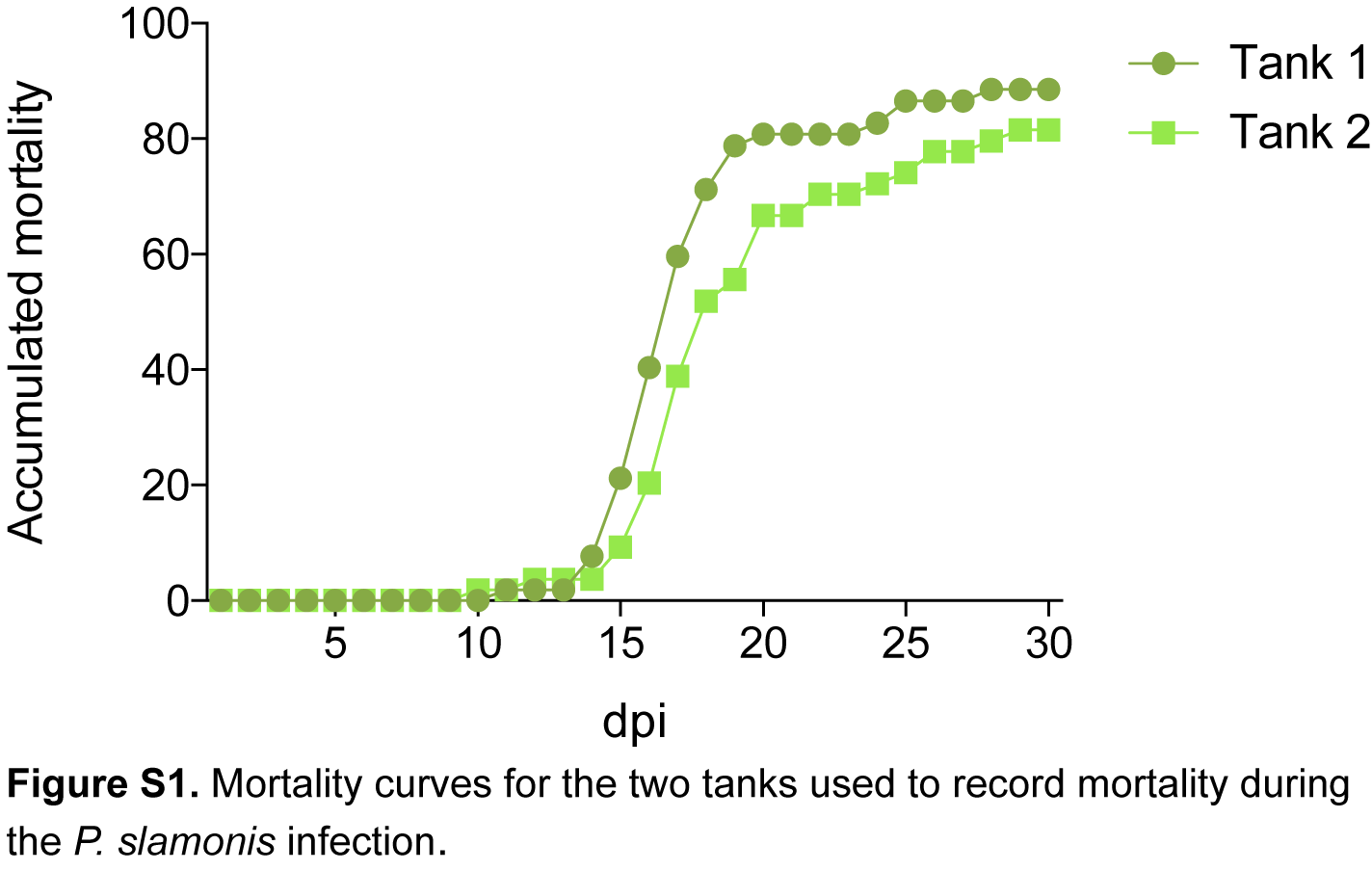

Supplement: Supplementary file 1 [file Image_1.TIF]

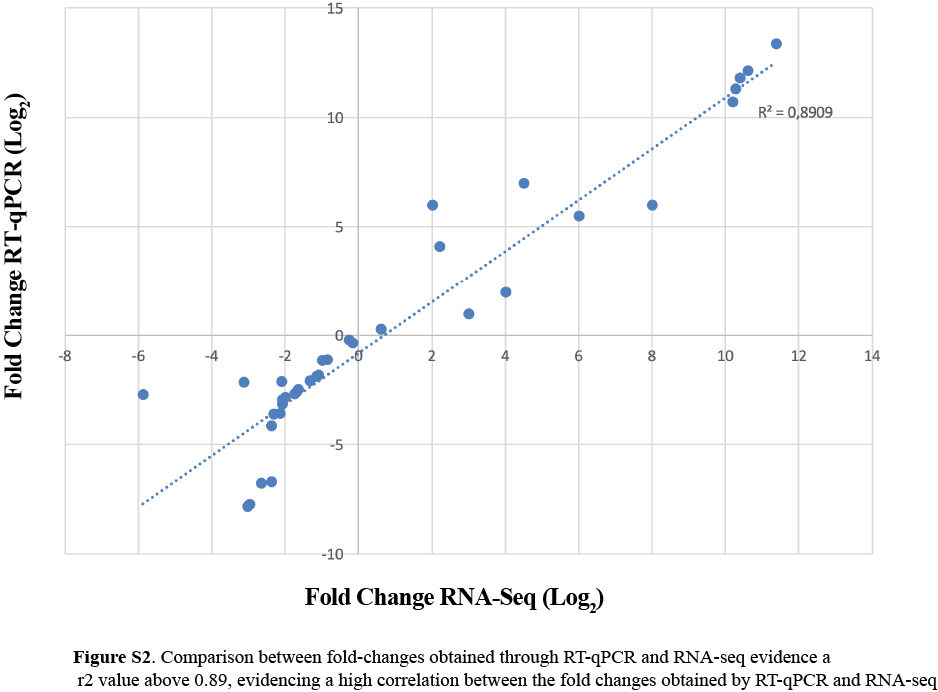

Supplement: Supplementary file 2 [file Image_2.PNG]

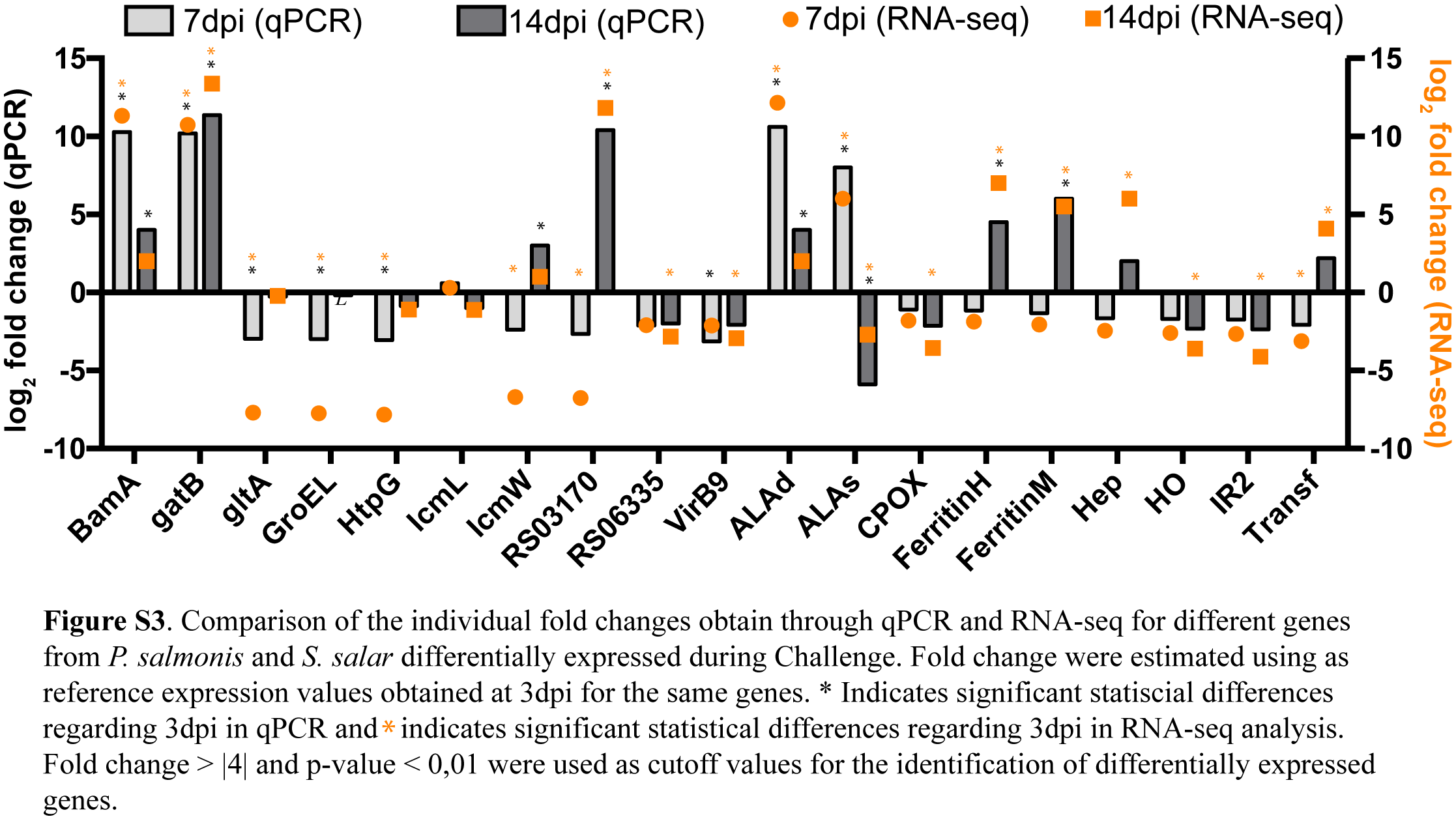

Supplement: Supplementary file 3 [file Image_3.TIF]
